# The triple overlap of COPD, severe obesity, and high risk of OSA: insights from an NHANES analysis

**DOI:** 10.1186/s12890-025-03752-4

**Published:** 2025-06-04

**Authors:** Staci L. Orbell, Jonna L. Morris, Paul W. Scott, Lynn M. Baniak, Christopher C. Imes, Bomin Jeon, Weiwen Wang, Yue Dong, Patrick J. Strollo, Faith S. Luyster

**Affiliations:** 1 Veterans Affairs Pittsburgh Healthcare System, Pittsburgh, PA USA; 2 School of Nursing, University of Pittsburgh, 3500 Victoria St, 415 Victoria Building, Pittsburgh, PA 15241 USA; 3 College of Nursing and Research Institute of Nursing Science, Seoul National University, Seoul, Korea; 4 Division of Pulmonary, Allergy, Critical Care Medicine and Sleep Medicine, Department of Medicine, University of Pittsburgh, Pittsburgh, PA USA

**Keywords:** Sleep apnea, Obstructive; pulmonary disease, Chronic obstructive; obesity, Morbid

## Abstract

**Purpose:**

With the rising prevalence of severe obesity, the coexistence of obstructive sleep apnea (OSA) and chronic obstructive pulmonary disease (COPD) often progresses to triple overlap syndrome, a condition with significant health implications. However, its prevalence remains poorly understood. Using population-based data from the National Health and Nutrition Examination Survey (NHANES), we examined the prevalence of triple overlap of COPD, severe obesity, and high risk for OSA (HR-OSA) and associated sociodemographic factors among US adults.

**Methods:**

A cross-sectional analysis was performed using NHANES data between 2005 and 2008 and 2015-March 2020. COPD diagnosis was collected via self-report questionnaire. HR-OSA was determined by an adapted Multivariable Apnea Prediction index. Severe obesity was defined as a body mass index of ≥ 40.0 kg/m^2^.

**Results:**

From 2005 to 2008 to 2015-March 2020, the proportion of participants with triple overlap of COPD, severe obesity, and HR-OSA increased from 0.653% (95% CI, 0.651-0.655%) to 1.560% (95% CI, 1.557-1.563%). During the same period, the increase in the age-standardized prevalence of severe obesity (from 6.298% [95% CI, 6.291-6.305%] to 8.943% [95% CI, 8.936-8.950%]) and HR-OSA (from 58.667% [95% CI, 58.646-58.688%] to 58.776% [95% CI, 58.758–58.794%]; ) exceeded the increase for COPD (from 9.223% [95% CI, 9.215-9.231%] to 10.213% [95% CI, 10.206-10.220%]). Women and those with low family income were more likely to have triple overlap of COPD, severe obesity, and HR- OSA.

**Conclusion:**

The triple overlap of COPD, severe obesity, and HR-OSA significantly increased among US adults over the past 15 years, with disparities across different sociodemographic groups.

**Trial registration:**

: Not applicable.

## Background

Chronic obstructive pulmonary disease (COPD) and obstructive sleep apnea (OSA) are highly prevalent respiratory disorders that are independent risk factors for cardiovascular morbidity and all-cause mortality [1–4]. The co-existence of these disorders, known as overlap syndrome, is common, with prevalence rates as high as 66%, depending on the population studied [5]. Overlap syndrome is characterized by more pronounced nocturnal oxygen desaturation, more frequent cardiovascular morbidity, and greater healthcare utilization compared to each disease alone [5, 6].

The prevalence of severe obesity (body mass index [BMI; calculated as weight in kilograms divided by height in meters squared], ≥ 40) among US adults has increased substantially over the past few decades [7, 8]. Disparities in severe obesity are evident across different sexes, racial/ethnic groups, and socio-economic statuses [7, 9]. It can be projected that the prevalence of severe obesity among overlap syndrome patients will increase and, consequently, more patients will present with “triple overlap” of COPD, OSA, and severe obesity, or triple overlap syndrome. In a small single-center study comparing overlap syndrome patients with and without obesity (BMI ≥ 30), those with overlap syndrome and obesity had an average BMI of 41.5 indicating severe obesity, were more likely to be female, had more severe OSA (i.e., higher apnea-hypopnea index, and more profound depth and duration of hypoxemia), and were more likely to require non-invasive ventilation [10]. Thus, triple overlap syndrome may represent a more severe phenotype at greater risk for poor outcomes and potential disparities in prevalence. No prior study has investigated the US nationwide prevalence trends and characteristics of triple overlap syndrome. In this study, we aimed to characterize trends in the prevalence of triple overlap of COPD, BMI ≥ 40 kg/m^2,^ and high risk for OSA (HR-OSA), among US adults overall and by sociodemographic subgroups using data from the National Health and Nutrition Examination Survey (NHANES) cycles 2005 to 2008 and 2015 to March 2020.

## Methods

### Study design

The NHANES uses a multistage probability sampling design to assess the health status of a nationally representative sample of the noninstitutionalized US civilian population. This cross-sectional analysis used data from the cycles that collected information on sleep apnea symptoms: 2005 to 2006, 2007 to 2008, 2015 to 2016, and the combined 2017 to March 2020. The National Center for Health Statistics (NCHS) recommends aggregating NHANES cycles whenever feasible [11], thus we combined data from the 2005 to 2006 and 2007 to 2008 cycles and from the 2015 to 2016 and 2017 to March 2020 cycles as protocols were identical. The NHANES protocols were approved by the NCHS Research Ethics Review Board, and written informed consent was obtained from all participants.

### Study population

The present analysis focused on adults aged 40 years and older, as COPD is considerably more prevalent in middle-aged and older adults [1], and those with complete data for demographics, medical conditions, sleep apnea symptoms, and BMI. COPD was defined by the presence of at least one of the following physician-diagnosed conditions: emphysema, chronic bronchitis, or COPD, which was assessed by the question, “Has a doctor or other health professional ever told you that you have [condition]?” High OSA risk was determined by an adaptation of the Multivariable Apnea Prediction (MAP) index [12] using NHANES variables, that has been described previously [13]. In addition, tailored cut points to MAP index scores based on sex and menopausal status were applied, as standard MAP scoring has been shown to underestimate women’s risk of OSA and given that postmenopausal women have a higher risk for OSA than premenopausal women [14]. Specifically, high OSA risk was defined as a MAP index score of 0.5 or greater in men [12], 0.154 in premenopausal women, and 0.375 in postmenopausal women. Application of the MAP index in this manner has shown to have high sensitivity for detecting OSA (84.2% for men, 90.5% for premenopausal women, and 67.9% for postmenopausal women) [15]. Menopausal status was determined using the reproductive health questionnaire. Postmenopausal was defined by a “no” response to the question “Have you had at least one menstrual period in the past 12 months?” and reported menopause status due to menopause or hysterectomy. Premenopausal was defined by a positive response to the above question or reported menopause status attributable to pregnancy, breastfeeding, or other medical conditions. Severe obesity was defined as a BMI of 40 or greater. Height and weight were measured in the NHANES Mobile Examination Center by a health technician. Triple overlap was defined as the presence of COPD, high OSA risk based on the MAP index, and severe obesity (i.e., BMI ≥ 40).

### Sociodemographic variables

Participants self-reported sociodemographic and clinical information on age, sex (male or female), race/ethnicity (non-Hispanic White, non-Hispanic Black, Hispanic, or other race and ethnicity [e.g., non-Hispanic Asian or multiple]), education (less than high school or high school or above), marital status (married or living with a partner, or single), family income, health insurance (uninsured or insured), and smoking status (never, former, or current). Based on family income and federal poverty guidelines, the family monthly poverty level index, a ratio of reported monthly income to poverty, was categorized as less than 130%, 130–349%, and 350% or greater.

### Statistical analysis

All analyses were conducted using SAS 9.4 (SAS Institute Inc.), with statistical significance set at 2-tailed *P* <.05, and incorporated recommended sample weights and accounted for clustering and stratification of the complex study design to ensure nationally representative parameter estimates [16, 17]. Taylor series linearization procedure on masked variance units from the demographic data was applied to estimate standard errors. Missing data were minimal, so cases with missing values were excluded, with post-stratification weights applied to help mitigate non-response bias.

Sociodemographic and clinical characteristics are presented, conditioned on cohort, as weighted means and percentages with 95% confidence limits. Rao-Scott χ2 and t-test were used to test differences in characteristics between cohorts for categorical and continuous variables, respectively. Prevalence estimates were age-standardized to the 2010 and 2020 US Census, for 2005–2008 and 2015-March 2020 cohorts respectively, in 3 age groups (40–49, 50–64, and 64 + years of age) using the direct method. Rate ratios were computed to assess relative changes in the age-standardized prevalence of triple overlap of COPD, severe obesity, and HR-OSA between the 2005–2008 and 2015-March 2020 cohorts, both overall and across various characteristics. Prevalence rates include 95% confidence intervals, using the normal distribution, while rate ratios use the lognormal distribution. Wald Z test was computed from the log rate ratio and standard error. Cohort-specific rates within each age stratum were presented, and absolute change was tested using Wald Z test based on rate difference and standard error with a short R function. Using the combined cohorts, binomial logistic regression was used to examine predictors of triple overlap. Odds ratios and 95% confidence intervals adjusted for age, sex, and race/ethnicity were produced.

## Results

### Participant characteristics

**Fig. 1:**
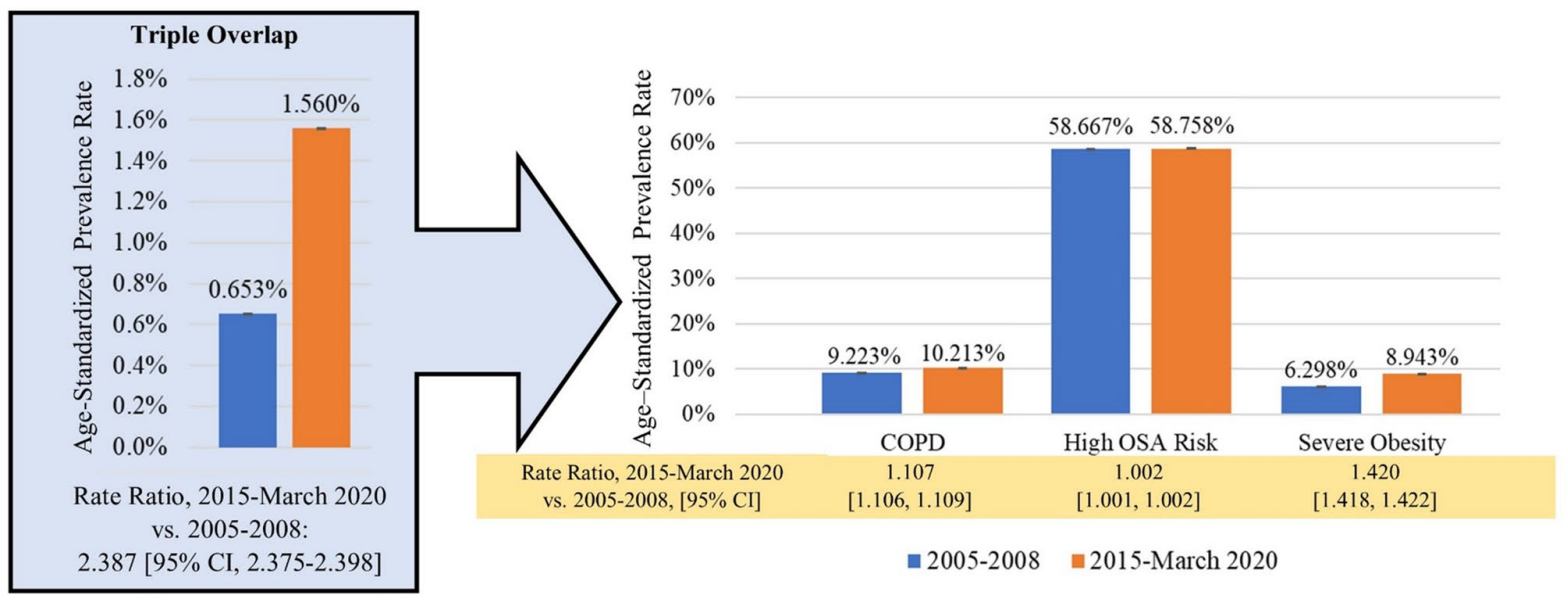
Age-Standardized Prevalance Rate of Triple Overlap of COPD, Severe Obesity, and High Risk of OSA, 2005-2008 vs. 2015-March 2020. Data are from the 2005 to 2008 and 2015 to March 2020 National Health and Nutrition Examination Survey (*N* = 16,356) and were weighted according to study guidelines. Abbreviations: COPD, chronic obstructive pulmonary disease; OSA, obstructive sleep apnea. Data on high OSA risk were missing for 687 participants

A total of 16,356 adults 40 years and older were included in this analysis, the demographics of whom are summarized in Table 1. Participants had a mean (SE) age of 58.0 (0.2) years, a mean (SE) BMI of 29.6 (0.1), 47.2% were male, and 70.7% were non-Hispanic White individuals. Between the 2005–2008 and 2015-March 2020 cohorts, mean age significantly increased (56.9 to 58.7 years, *P* <.01). Increases were also noted in the percentage of individuals classified as “other” race, high school or above level of education, insured individuals, and among individuals living < 130% of the federal poverty line (Table 1). Age-standardized rates of COPD (9.223% [95% CI, 9.215–9.231%] to 10.213% [95% CI, 10.206–10.220%]; *P* <.01), high OSA risk (58.667% [95% CI, 58.646–58.688%] to 58.776% [95% CI, 58.758–58.794%]; *P* <.01), and severe obesity (6.298% [95% CI, 6.291-6.305%] to 8.943% [95% CI, 8.936-8.950%]; *P* <.01) significantly increased from the 2005–2008 cohort to the 2015-March 2020 cohort (Fig. 1). Severe obesity corresponded to a 42% relative increase in prevalence from the 2005–2008 cohort to the 2015-March 2020 cohort, while COPD had an 11% relative increase and high risk OSA only increased by 0.2%.

**Table 1:** Weighted characteristics of US adults 40 years or older in the 2005–2008 and 2015-March 2020 NHANES cycles

	Weighted % (95% CI)^a^ (*N* = 16,356)			
Characteristic	Combined Cohorts (*N* = 16,356)	2005–2008 (*n* = 6,797)	2015-March 2020 (*n* = 9,559)	***P*** value
Age, mean (SE) [95% CI], y	58.0 (0.2) [ 57.5–58.4]	56.9 (0.4) [56.1–57.7]	58.7 (0.3) [58.1–59.3]	< 0.01
Age group, y				
40–49	29.5 (28.0-31.1)	33.8 (31.2–36.5)	26.6 (24.7–28.4)	< 0.01
50–64	40.6 (39.4–41.9)	39.5 (37.8–41.1)	41.4 (39.6–43.3)	
≥ 65	29.8 (28.3,31.4)	26.7 (24.5–29.0)	32.0 (30.0–34.0)	
Sex				
Men	47.2 (46.4–48.1)	47.2 (45.9–48.5)	47.3 (46.2–48.3)	0.93
Women	52.8 (51.9–53.6)	52.8 (51.5–54.1)	52.7 (51.6–53.9)	
Menopausal status				
Premenopausal	42.4 (40.0-44.8)	65.8 (62.2–69.4)	26.6 (24.3–28.9)	< 0.001
Postmenopausal	57.6 (55.2–60.0)	34.2 (30.6–37.8)	73.4 (71.1–75.7)	
Race and ethnicity				
Non-Hispanic White	70.7 (67.7–73.8)	75.6 (71.4–79.9)	67.6 (63.2–71.5)	< 0.01
Non-Hispanic Black	10.6 (8.9–12.4)	10.6 (7.9–13.3)	10.7 (8.3–13.0)	
Hispanic	11.2 (9.5–12.8)	8.7 (6.8–10.6)	13.0 (10.5–15.4)	
Other^b^	7.4 (6.4–8.5)	5.2 (3.9–6.4)	9.0 (7.4–10.6)	
Education level^c^				
High school or less	15.7 (14.3–17.0)	19.4 (17.2–21.6)	13.1 (11.5–14.8)	< 0.01
High school or above	84.3 (83.0-85.7)	80.6 (78.4–82.8)	86.9 (85.3–88.5)	
Insurance status^d^				
Insured	89.4 (88.3–90.6)	87.3 (85.3–89.4)	90.8 (89.5–92.2)	< 0.01
Uninsured	10.6 (9.4–11.7)	12.7 (10.6–14.7)	9.2 (7.9–10.5)	
Income-poverty ratio				
<130% of FPL	29.2 (27.4–31.0)	23.9 (21.9–25.9)	32.8 (30.2–35.5)	< 0.01
130-349% of FPL	33.2 (31.7–34.7)	35.5 (33.5–37.5)	31.6 (29.5–33.7)	
≥350% of FPL	37.6 (35.5–39.8)	40.6 (37.4–43.7)	35.6 (32.8–38.5)	

Abbreviations: FPL, federal poverty level; NHANES, National Health and Nutrition Examination Survey

^a^Percentages were adjusted for NHANES survey weights

^b^Other race and ethnicity (e.g., non-Hispanic Asian American or multiple races or ethnicities)

^c^Data were missing for 27 participants

^d^Data were missing for 26 participants

### Age-standardized prevalence of and factors associated with triple overlap of COPD, severe obesity, and HR-OSA

Between 2005 and 2008 and 2015-March 2020, there was a significant increase in the proportion of adults with triple overlap of COPD, severe obesity, and HR-OSA (0.653%; 95% CI, 0.651%-0.655–1.560%; 95% CI, 1.557-1.563%; *P* <.01), with a nearly 2.4-fold relative increase (95% CI, 2.375–2.398) (Fig. 1). Prevalence of this triple overlap increased significantly across all sociodemographic groups (Table 2), with the greatest increment observed for adults aged 50–64 years, males, Hispanic people, and those who were uninsured. Among all US adults in the 2005–2008 and 2015-March 2020 NHANES cycles, there was a higher likelihood of having triple overlap of COPD, severe obesity, and HR-OSA for women relative men while controlling for age and race/ethnicity (Table 3). Adults who had an income-poverty ratio of 130-349% and ≥ 350% of the federal poverty line were less likely to have this triple overlap than adults with an income-poverty ratio less than 130% of the federal poverty line adjusting for sex, age, and race/ethnicity.

**Table 2:** Estimated prevalence of triple overlap of COPD, severe obesity, and high risk for OSA among US adults 40 years or older from 2005–2008 and 2015-March 2020 by sociodemographic characteristics

	Estimated age-standardized prevalence of triple overlap, % (95% CI)^a^			
Characteristic	2005–2008 (*n* = 6,797)	2015-March 2020 (*n* = 9,559)	***P*** value	Rate Ratio, 2015-March 2020 vs. 2005–2008 (95% CI)
Age group, y				
40–49	0.750 (0.746–0.753)	1.049 (1.044–1.053)	< 0.01	1.399 (1.390–1.409)
50–64	0.520 (0.517–0.523)	2.089 (2.084–2.094)	< 0.01	4.014 (3.989–4.040)
≥ 65	0.830 (0.825–0.835)	1.091 (1.087–1.095)	< 0.001	1.314 (1.305–1.323)
Sex				
Men	0.314 (0.312–0.316)	1.210 (1.207–1.214)	< 0.01	3.852 (3.824–3.879)
Women	0.989 (0.985–0.993)	1.914 (1.910–1.919)	< 0.01	1.934 (1.927–1.946)
Race and ethnicity				
Non-Hispanic White	0.613 (0.611–0.616)	1.608(1.604–1.612)	< 0.01	2.625 (2.611–2.632)
Non-Hispanic Black	1.507 (1.500-1.517)	1.559 (1.551–1.568)	< 0.01	1.035 (1.026–1.044)
Hispanic	0.218 (0.213–0.223)	1.289 (1.282–1.296)	< 0.01	5.914 (5.780–6.050)
Other^b^	0(--)^c^	1.852 (1.841–1.863)	NA	NA
Education level				
High school or less	0.928 (0.921–0.934)	2.040 (2.030–2.049)	< 0.01	2.199 (2.180–2.218)
High school or above	0.627 (0.624–0.629)	1.491 (1.488–1.494)	< 0.01	2.381 (2.370–2.387)
Insurance status				
Insured	0.737 (0.735–0.740)	1.555 (1.552–1.558)	< 0.01	2.110 (2.101–2.119)
Uninsured	0.105 (0.103–0.107)	1.488 (1.479–1.497)	< 0.01	14.085 (13.889–14.493)
Income-poverty ratio				
<130% of FPL	1.284 (1.278–1.291)	2.373 (2.367–2.380)	< 0.01	1.848 (1.838–1.859)
130-349% of FPL	0.481 (0.478–0.484)	1.591 (1.586–1.597)	< 0.01	3.309 (3.284–3.333)
≥350% of FPL	0.458 (0.455–0.461)	0.830 (0.826–0.833)	< 0.01	1.812 (1.799–1.825)

Abbreviations: COPD, chronic obstructive pulmonary disease; FPL, federal poverty level; OSA, obstructive sleep apnea

^a^Percentages were adjusted for NHANES survey weights

^b^Other race and ethnicity (e.g., non-Hispanic Asian American or multiple races or ethnicities)

^c^There were no adults classified as “other race” who met eligibility criteria for triple overlap in this cohort

**Table 3:** Adjusted odds ratios for factors associated with triple overlap of COPD, severe obesity, and high risk for OSA in NHANES from 2005–2008 and 2015-March 2020 (Total sample = 16,356)

Characteristic	Odds ratio (95% CI)
Age group, y	
40–49	1 [Reference]
50–64	1.614 (0.960, 2.715)
≥ 65	1.057 (0.581, 1.920)
Sex	
Men	1 [Reference]
Women	1.660 (1.003, 2.748)
Race and ethnicity	
Non-Hispanic White	1 [Reference]
Non-Hispanic Black	1.272 (0.876, 1.847)
Hispanic	0.773 (0.444, 1.346)
Other^a^	1.014 (0.534, 1.927)
Education level	
High school or less	1 [Reference]
High school or above	0.824 (0.510, 1.330)
Insurance status	
Insured	1 [Reference]
Uninsured	0.733 (0.449, 1.196)
Income-poverty ratio	
<130% of FPL	1 [Reference]
130-349% of FPL	0.559 (0.334, 0.937)
≥350% of FPL	0.312 (0.186, 0.524)

Abbreviations: COPD, chronic obstructive pulmonary disease; FPL, federal poverty level; NHANES, National Health and Nutrition Examination Survey; OSA, obstructive sleep apnea

^a^Other race and ethnicity (e.g., non-Hispanic Asian American or multiple races or ethnicities)

## Discussion

The findings of this nationally representative survey study indicate that the age-standardized prevalence of triple overlap of COPD, severe obesity, and HR-OSA increased 2.4 times between 2005 and 2008 and 2015-March 2020, with disparities across different sociodemographic groups. After accounting for age, sex, and race/ethnicity, adults aged 50–64, women, and those with an income-poverty ratio below 130% of the federal poverty line were more likely to experience this triple overlap. To our knowledge, this is the first study to examine the prevalence of the triple overlap of COPD, severe obesity, and HR-OSA, which may serve as a proxy for diagnosed triple overlap syndrome.

The most recent estimated prevalence rate of triple overlap of COPD, severe obesity, and HR-OSA (i.e., 2015-March 2020 data) found in our study was 1.6%. Prior studies in general and hospital populations reported that the prevalence of overlap syndrome ranged from 1 to 3.6% [18]. The significant increase in the prevalence of severe obesity as noted in our study (6.3–8.9%) and consistent with previous reports may have contributed, in part, to the increased prevalence of triple overlap over time [7, 8]. Obesity is a primary risk factor for OSA and significantly increases the risk of OSA in the COPD population [19], making patients with overlap syndrome more likely to be phenotypically obese [20]. In COPD populations, patients who have overweight and obesity have reduced all-cause and cardiovascular mortality compared to patients with normal weight, referred to as the “obesity paradox” [21, 22]. This protective effect diminishes with severe obesity in this population [23]. Similarly, an increased risk for nonfatal cardiovascular events, including myocardial infarction and stroke, has been observed in patients with COPD who have severe obesity compared to patients with normal weight [22]. Thus, the obesity paradox may not apply to those with triple overlap syndrome. As compared to those without obesity, overlap syndrome patients with obesity (average BMI of 41.5, indicating severe obesity) have been shown to exhibit more severe OSA and deeper, more prolonged hypoxemia, which predict cardiovascular morbidity and mortality [4, 24, 25]. These findings underscore the complex interplay between severe obesity, OSA, and COPD in triple overlap syndrome, highlighting the need for targeted interventions to mitigate cardiovascular disease risk in this vulnerable population.

The overall rise in the prevalence of triple overlap of COPD, severe obesity, and HR-OSA should be considered within the context of sex differences and socioeconomic disparities across different populations. We observed that the highest-risk groups for this triple overlap are women when controlling for age and race/ethnicity, and individuals living in poverty when controlling for age, sex, and race/ethnicity. These findings are consistent with a substantial body of research demonstrating a strong link between poverty and obesity [26]. Socioeconomic challenges may limit access to healthy food options and opportunities for regular exercise, making it more difficult to maintain a healthy lifestyle. The menopausal transition increases the risk for both weight gain [27, 28] and OSA [14], compounding the risk of developing triple overlap syndrome. Among NHANES participants, there was a significant increase in the rate of postmenopausal women from the 2005–2008 cohort to the 2015-March 2020 cohort, which may have contributed to the increase in triple overlap of COPD, severe obesity, and HR-OSA over time. By applying specific MAP index criteria to accurately classify women by menopausal status, we identified midlife women as a particularly high-risk group for this triple overlap. Future research should explore the unique risk factors affecting midlife women, with particular attention to the menopausal transition and the influence of socioeconomic disadvantage, to help clarify their heightened vulnerability to triple overlap syndrome.

The clinical relevance of identifying this high-risk population has important implications regarding treatment. These patients are likely to have significant restrictive lung disease leading to carbon dioxide retention resulting from hypoventilation [29]. Standard approaches to treating OSA with continuous positive airway pressure or bilevel positive airway pressure are often configured for comfort as opposed to ventilatory strategy. Concomitant COPD increases the risk of tracheobronchitis and subsequent lower airway infection [30]. In addition, chronic hypercapnia can negatively impact immune function, increasing the propensity for pneumonia [31]. If chronic respiratory failure related to triple overlap syndrome is not recognized and effectively treated, these patients are at high risk for hospital admission for acute on chronic respiratory failure, along with the associated increased healthcare costs, morbidity, and mortality. Furthermore, low socioeconomic status is associated with poor health literacy and challenges to health care access [32]. These factors place the triple overlap syndrome population at differential risk for adverse health outcomes if not effectively addressed.

### Limitations

This study has several limitations. These data used for this secondary analysis is cross-sectional from NHANES, the nature of which precludes inference of causal relationships. Further, these data were derived from participants’ self-report, which may be prone to errors in recall, including a self-report of the variables used to classify a diagnosis of COPD and responses to sleep-related questions used to calculate OSA risk. It is unknown the extent to which participants in this study had diagnosed OSA as these data were not available through NHANES, thus we relied on OSA risk as calculated using a validated instrument, tailored by sex and menopausal status. However, this approach has not been previously validated using NHANES data. Finally, although adjacent NHANES cycles were combined for this analysis, the unweighted sample size of adults with triple overlap of COPD, severe obesity, and HR-OSA between the two cohorts was relatively small, which limits the statistical power needed to detect small to moderate changes in sociodemographic characteristics over time, particularly in adjusted models.

## Conclusions

Our findings reveal a significant rise in the prevalence of triple overlap of COPD, severe obesity, and HR-OSA over a 15-year period in the US, with severe obesity as a primary driver. Disparities were notable among women and those with low family income. Future research should examine the intersection of obesity and structural determinants of health inequities to inform a targeted clinical approach that improves outcomes for high-risk groups.

## Data Availability

All NHANES data and information are publicly available at https://www.cdc.gov/nchs/nhanes/index.htm.

## Abbreviations

BMI: Body mass index
COPD: Chronic obstructive pulmonary disease
HR: OSA-High risk for OSA
MAP: Multivariable Apnea Prediction
NCHS: National Center for Health Statistics
NHANES: National Health and Nutrition Examination Survey
OSA: Obstructive sleep apnea
